# Abnormalities of hippocampus and frontal lobes in heart failure patients and animal models with cognitive impairment or depression: A systematic review

**DOI:** 10.1371/journal.pone.0278398

**Published:** 2022-12-09

**Authors:** Ziwen Lu, Yu Teng, Lei Wang, Yangyang Jiang, Tong Li, Shiqi Chen, Baofu Wang, Yang Li, Jingjing Yang, Xiaoxiao Wu, Weiting Cheng, Xiangning Cui, Mingjing Zhao

**Affiliations:** 1 Key Laboratory of Chinese Internal Medicine of Ministry of Education and Dongzhimen Hospital, Beijing University of Chinese Medicine, Beijing, China; 2 Department of Cardiovascular, Guang’anmen Hospital, China Academy of Chinese Medical Sciences, Beijing, China; Kurume University School of Medicine, JAPAN

## Abstract

**Aims:**

This systematic review aimed to study the hippocampal and frontal changes of heart failure (HF) patients and HF animal models with cognitive impairment or depression.

**Methods:**

A systematic review of the literature was conducted independently by reviewers using PubMed, Web of Science, Embase, and the Cochrane Library databases.

**Results and conclusions:**

30 studies were included, involving 17 pieces of clinical research on HF patients and 13 studies of HF animal models. In HF patients, the hippocampal injuries were shown in the reduction of volume, CBF, glucose metabolism, and gray matter, which were mainly observed in the right hippocampus. The frontal damages were only in reduced gray matter and have no difference between the right and left sides. The included HF animal model studies were generalized and demonstrated the changes in inflammation and apoptosis, synaptic reduction, and neurotransmitter disorders in the hippocampus and frontal lobes. The results of HF animal model studies complemented the clinical observations by providing potential mechanistic explanations of the changes in the hippocampus and frontal lobes.

## Introduction

Heart failure (HF) is a rapidly increasingly cardiovascular disease with high morbidity and mortality around the world over the past decade [1]. Numerous researches have investigated that cognition impairment (CI) and depression were prevalent in patients with HF [2, 3], with the incidence of approximately 43–82% and 30%, respectively [3, 4]. HF and cognitive impairment/depression comorbidity increase hospitalization and risk of Alzheimer’s disease, impacting the living quality in HF patients [5].

The cognitive declines in HF patients were mild cognitive impairment (MCI) affecting memory, attention, and executive function, which was different from Alzheimer’s disease [6]. It has been known that the neurological symptoms were closely bound up with the injury of the brain’s specific regions [7, 8]. Hippocampus is related to memory and is also seen as the regulator of emotion [9, 10]. Frontal lobe is critical to acquisition, execution, and control of a wide range of functions, from basic motor response to complex decision-making [11]. The function of the hippocampus and frontal lobe was consistent with cognitive impairment in HF patients, therefore, this review focused on changes in these two regions. Moreover, it has been reported fragmentary studies about imaging of the hippocampus and frontal lobe after HF, however, it lacks systematic summaries and generalizations about damage changes of the hippocampus and frontal lobe after HF. Meanwhile, the animal models could complement the clinical observations by providing potential mechanistic explanations. Therefore, the characteristic changes of the hippocampus and frontal lobe in HF animal model still need to be systematically reviewed.

This systematic review screened the current literature to identify characteristic cognitive impairment and depression in HF. Most importantly, it discussed the changes in the hippocampus and frontal lobe in HF patients, and thus whether these changes had differences between the right and left sides. Then, pathological mechanisms of hippocampal and frontal damage in HF animal model studies were also summarized to complement clinical observations. This systematic review would provide a reference for future clinical prevention and treatment of HF.

## Methods

The report of this systematic review was prepared based on the PRISMA 2020 Statements.

### 1. Information sources and search strategy

The published articles were searched comprehensively in electronic databases (PubMed, Web of Science, Embase, and Cochrane Library) up to August 2022. Keywords and Medical Subject Heading (MeSH) terms used in these searches included heart failure, heart decompensation, myocardial failure, congestive heart failure, cognitive dysfunction, cognitive impairment, cognitive disorder, mental deterioration, depression, depressive symptoms, emotional depression, hippocampus, hippocampus propers, hippocampal formation, ammon horn, subiculum, frontal lobes, frontal cortex, supplementary eye field, and brodmann Area 8. No filter or limitation was used during the search. The detailed search strategy was acquired in supplement materials. For all identified studies, a manual search was conducted of their references and review articles to locate additional relevant studies.

### 2. Inclusion and exclusion criteria

The inclusion criteria of the clinical studies were as follows: (1) Participants: Patients meting the diagnostic criteria of HF were included, with or without the control group; (2) Method of research: Cohort studies, case-control studies, cross-sectional studies, two-group comparative studies and prospective studies were included; (3) The study had to involve in the structure and function of the hippocampus and frontal lobes.

The inclusion criteria of the animal experiments studies were as follows: (1) Animals were modeled for heart failure or myocardial infarction (MI) in vivo experiment; (2) The researches pointed out the structure and function of the hippocampus and frontal lobes; (4) Languages were not restricted and the literatures should be published in the official journals.

The exclusion criteria were as follows: (1) Articles with incomplete information; (2) Reviews, meta-analysis, and corresponding/conference abstracts; (3) Comorbidities in clinical patients include neurological diseases such as stroke, Alzheimer’s Disease (AD), protopathy of the brain and other neurological symptoms.

### 3. Study selection and data extraction

Titles and abstracts of all studies were assessed independently by two researchers (ZW.L. and Y.T.) according to the inclusion and exclusion criteria. Firstly, duplicate literature that came from different databases were removed from the initial results. Secondly, distinctly irrelevant literature were eliminated via titles and abstracts. Thirdly, we screened the full texts and finally confirmed the included studies.

Two authors (ZW.L. and Y.T.) individually extracted data from the included literature employing a standardized sheet prepared for this review. Data on clinical studies included clinical characteristics of the study population were collected, it consists of first author’s name, year of publication, country, sample size, age, HF severity and comorbidities; cognitive function and psychological test; changes and damage in the hippocampus as well as relevant hippocampal region and changes of frontal lobes. As for animal researches, the basic information of included experimental studies was extracted, including animal species, sex, weight, age, sample size, etc. In addition, we also acquired the cognition and depression tests and characteristics of hippocampal and frontal lobes changes. Any disagreement was resolved by discussing and consulting with the corresponding authors (MJ.Z.).

### 4. Assessment of bias risk and study quality

Newcastle-Ottawa scales (NOS) was used to assess the quality of clinical studies based on three factors: the selection of research population, compatibility of the study groups, and measurement of exposure factors. Each study scored 0–9 points. Cochrane risk of bias tool was used to assesses clinical studies as low or high risk for the following forms of bias: selection, performance, detection, attrition, reporting, and other.

SYRCLE’s risk of bias tool was applied to assess the quality of animal researches, a total of ten items of six projects were used as evaluation criteria including selection bias, performance bias, detection bias, attrition bias, reporting bias, and other sources of bias. Each item was evaluated as “high risk”, “low risk”, or “unclear” [12].

### 5. Summary analysis

Due to the high heterogeneity of the included literature, we only compared different trends and mechanisms in the hippocampus change both in clinical and animal researches. Therefore, a qualitative synthesis was adopted for this systematic review.

## Results

### 1. Overview of the studies

#### 1.1 Search results

**Fig 1:**
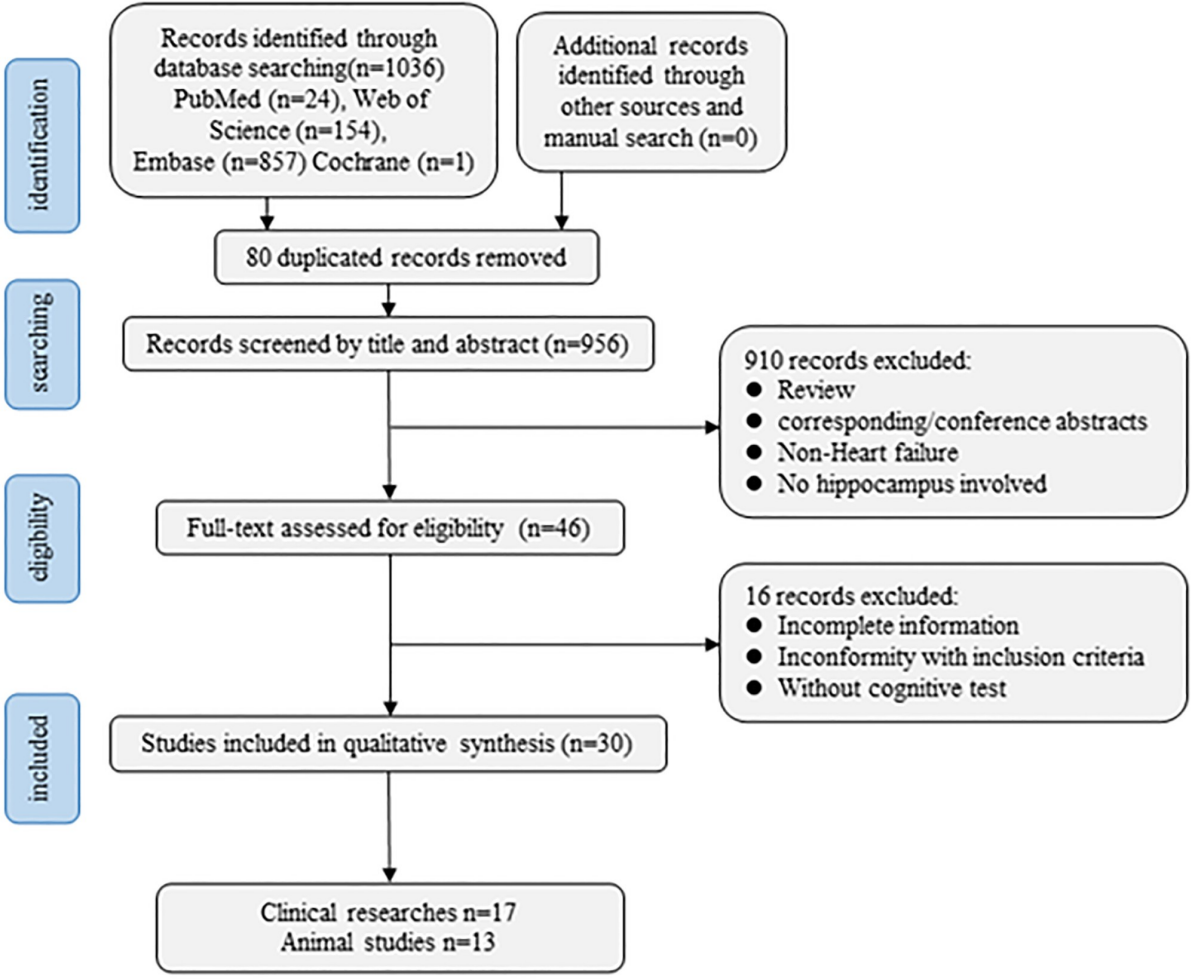
Flowchart of the study selection process.

The process of literature selection, including identification, screening, eligibility and included, are described in Fig 1. Of the 1036 articles in search of PUBMED, Embase, Web of Science, and Cochrane Library, 80 were excluded because of duplication, and 910 were excluded after reading titles and abstracts. Then 16 articles were excluded according to the full text and finally 30 studies (17clinical researches+13 animal studies) were included in this review (shown in Fig 1).

### 1.2 Characteristics of clinical researches

As shown in Table 1, among the included 17 clinical studies, seven studies were conducted in America and four studies were in Japan, while the remaining studies were based in Germany (2/17), Brazil (1/17), China (1/17), Australia (1/17) and Poland (1/17). There were ten case-control studies, three cross-sectional study, one prospective study, two cohort studies, and one two-group comparative study. The sample sizes of the 17 studies ranged from 14 to 491 and most of them included males and females. The mean average age ranged from 12.9 to 84. The severity of HF for patients was assessed by the New York Heart Association (NYHA) classification for HF, left ventricular ejection fraction (LVEF), and the N-terminal prohormone of brain natriuretic peptide (NT-proBNP). Thirteen studies included HF patients with NYHA II to Ⅳ, while four of them also contained patients with NYHA Ⅰ. The remaining studies only reported the LVEF value of patients with a range of 14% to 51.3%. According to ACC/AHA (American College of Cardiology/American Heart Association) stages of HF [13], fourteen studies included Stage C HF patients, and two studies included both Stage B and C HF patients. One study included Stage B HF patients. Most of the studies showed the comorbidities of the subjects.

**Table 1:** Summary of clinical study characteristics.

Author, year	Country	Study design	Sample size	Mean age	LVEF (%)	NYHA class	NT-proBNP level(pg/ml)	Comorbidities	ACC/AHA Stages of HF
Roy, B 2017 [18]	America	case-control study	•Control group: 29 (healthy subjects) •HF group: 19	51.4 55.5	Control:—HF:30.5±11.5	II (80%) III (20%)	N/A	Hypertensive (12) atrial fibrillation (4) type 2 diabetes (5)	Stage C
Mueller, K 2020 [33]	Germany	case-control study	• Control group: 60 (healthy subjects) •NAD group: 22 •CAD- group: 20 •CAD+ group: 35	54.9	Control:— NAD:62.5±5.4 CAD-:63.7±5.4 CAD+:47.2±11.7	N/A	NAD:68.8 CAD-:66.7 CAD+:2758.8	Arterial hypertension (50) Diabetes (18) Smoking (57)	Stage B
Pan, A 2013 [20]	America	two-group comparative study	•Control group: 50 (healthy subjects) •HF group: 17	50.6 54.4	Control:— HF:28±7	II (*n* = 17)	N/A	Type II diabetes (4) atrial fibrillation (2) hypertension (6)	Stage C
T AN I A C. T. F. A L V E S, 2006 [16]	Brazil	cross-sectional design	•Control group: 18 (healthy subjects) •MDD-HF group:17 •Non-depressed HF group: 17	72.8 76.0 73.7	Controls: 73.4±4.2 MDD-HF: 35.5±7.6 Non-depressed HF:39.8±3.3	II (*n* = 18) III (*n* = 16)	N/A	atrial fibrillation (5) cigarette smoking (12) diabetes (20)	Stage C
Suzuki, H 2016 [9]	Japan	case-control study	•Stage B group: 40 •Stage C group: 40	65.0 66.8	Stage B: 59.6±14.7 Stage C: 43.1±17.5	N/A	N/A	(StageB/StageC) Hypertension (52.5%/65%) Diabetes (35%/20%) Smoking (70%/57.5)	Stage B Stage C
Frey, A 2021 [15]	Germany	cohort study: follow up 1 and 3 years	148 mild stable HF patients	64.5	Mean LVEF: 43.6±8.1	I (*n* = 41) II (*n* = 88) III (*n* = 19)	N/A	Myocardial infarction (54.1%) Atrial fibrillation (19.6%) Hypertension (79.7%) Diabetes mellitus (29.1%) Renal dysfunction (35.8%)	Stage C
Suzuki, H 2020 [21]	Japan	cohort study: follow up 3.1 ± 0.5 years	70 CHF patients	65.0	Mean LVEF: 51.3±16.9	N/A	107.5±127.5	Ischemic heart failure (54.3%) Hypertension (64.3%) Diabetes (30%)	Stage B Stage C
Niizeki, T 2019 [29]	Japan	prospective study	491 CHF patients	84	Mean LVEF: 52±15	I (*n* = 32) II (*n* = 214) III (*n* = 205) Ⅳ(*n* = 40)	421	Hypertension (151/286) Diabetes mellitus (43/105) Hyperlipidemia (74/149) Atrial fibrillation (94/160)	Stage C
Woo, M. A 2015 [30]	America	case-control study	•Control group: 34 (healthy subjects) •HF group: 17	52.3 54.4	Control:—HF:28.3±6.8	II (94%) III (6%)	N/A	N/A	Stage C
Yun, M 2020 [37]	China	case-control study	•Control group:55 (healthy subjects) •HF group: 102	56.3	Control:— HF:15–37.3	III--Ⅳ (*n* = 50)	>100	Hypertension (36.3%) Diabetes (32.3%) Dyslipidemia (21.6%) Prior and current smokers (76.4%)	Stage C
Woo, M. A 2005 [32]	America	case-control study	•Control group:16 (healthy subjects) •HF group: 6	48 49	N/A	III-IV (*n* = 6)	N/A	Sinus rhythm(*n* = 5) Chronic atrial fibrillation(*n* = 1)	Stage C
Wykrętowicz, A 2019 [31]	Poland	case-control study	•Control group:38 (healthy subjects) •HF group: 25	62 64	Control: 64 HF:32	N/A	N/A	Hypertension (*n* = 10) Diabetes (*n* = 6)	Stage C
Menteer, J 2010 [34]	America	case-control study	•Control group:7 (healthy subjects) •HF group: 7	12.9	Control: -- HF:14±3	II (*n* = 2) III (*n* = 3) Ⅳ(*n* = 2)	N/A	N/A	Stage C
Woo, M. A 2009 [35]	America	case-control study	•Control group:13 (healthy subjects) •HF group: 49	50.6 54.6	Control: -- HF:28±7	II (*n* = 4)	N/A	Sinus rhythm (85%) Atrial fibrillation (15%)	Stage C
Almeida, O. P 2012 [17]	Australia	cross-sectional study	•Control group:64 (healthy subjects) •HF group: 35	68.7 69.2	Control: 68.1±5.2 HF:30.4±7.8	I (31.4%) II (54.3%) III (14.3%)	Control: 59.4±70.5 HF:233.8±194.5	Smokers (68.6%)	Stage C
Park, B 2016 [22]	America	case-control study	•Control group:53 (healthy subjects) •HF group: 27	53	Control: -- HF:28.0±9.2	II (100%)	N/A	N/A	Stage C
Ichijo, Y. 2020 [19]	Japan	cross-sectional study	•Control group:35 (healthy subjects) •HF group: 28	70.5 70.6	Control: -- HF:24	I (60%) II (40%)	Control: 50.8 HF:346.6	Hypertension (60%) Diabetes mellitus (54%) Dyslipidemia (77%) Atrial fibrillation (31.4%)	Stage C

Abbreviations: **HF** = heart failure; **NYHA** = New York Heart Association; **LVEF** = left ventricular ejection fraction; **Stage C patients** = who had past or current CHF symptoms; **Stage B patients** = who had structural heart disease but had never had CHF symptoms; **CAD+** = coronary artery disease with heart failure; **CAD-** = coronary artery disease with sufficient heart function; **NAD** = no abnormality detected; **z-scores** = refers to the measured value of a growth index of the tested population and reference to the average value of this index and the ratio of the overall standard deviation of this index. The z-score in patients with cardiac events was significantly higher than that in patients without cardiac events. **MDD-HF** = major depressive disorder-heart failure; **NT-proBNP** = N-terminal prohormone of brain natriuretic peptide.

#### 1.3 Characteristics of animal studies and assessment of HF model

Table 2 presents the data of experimental studies on authors, animal race, sex, age or weight, and sample size. Among the 13 experimental studies, 11 studies reported that the HF model was made by ligating the LAD coronary, and the surgery of transverse aortic constriction and arteriovenous fistula were made to build the HF model in the remaining two studies. The feeding time after surgery of all experimental studies ranged from 6 hours to 16 weeks. To evaluate the cardiac function of animals, thirteen studies presented a significant decrease in EF and FS, while there was no change in EF of one study [10]. In addition, there was only one study recording the infarct size (50.25 ± 1.85%) of the heart [14].

**Table 2:** General characteristics information and cardiac function of experimental studies.

Author, year	Sex/Animal strain	Weight(g) or Age(month)	Sample size	Model (Methods)	Time	Cardiac function assessments
Shinoda, 2016 [38]	Male mice	10 weeks	15	HF(TAC)	6 weeks	FS, LVESD, LVEDD↓;
Lijun zhang, 2019 [39]	Male mice	8–10 weeks	30	HF(LAD)	6 weeks	LVEF<40%; BNP↑
C. Liu, 2014 [14]	Male rats	250–300 g	18	MI(LAD)	6 hours	infarct size was 50.25 ± 1.85%
Frey, 2014 [24]	Male mice	6–9 weeks	29	HF(LAD)	8 weeks	FS↓; ESD, EDD↑; ESA,EDA↑
Koji Ito, 2013 [66]	Male mice	10 weeks	30	MI(LAD)	1 or 4 weeks	LV dimensions↑; FS↓
Y. Zhou, 2020 [40]	Female rat	12 weeks	14	HF(LAD)	8 weeks	LVIDd, LVIDs, EDV and ESV↑; EF, FS, IVSd, IVSs↓
Yingbin Ge, 2020 [41]	Male mice	20–25 g	30	HF(LAD)	2 weeks	LVEF↓; LVFS↓
Austin T. H. Duong, 2019 [25]	Male mice	8 weeks	/	HF(LAD)	8 weeks	LVIDd, (LVIDs) dimension↑; LVEF↓; LVFS↓
Camilo Toledo, 2019 [10]	Male rat	250 g	24	Volume overload HF (Surgical creation of an arteriovenous fistula)	8 weeks	EDD, EDV, ESD,ESV↑; MABP ↓; EF no change
H. Suzuki, 2015 [36]	Male rat	9 weeks	53	HF(LAD)	16 weeks	FS ↓; LVDd, PWTd↑
Kim Lee, 2017 [26]	Male rat	250–300 g	32	HF(LAD)	10 weeks	LVESD,LVEDD↑; LVEF↓
Md Rezaul Islam, 2021 [28]	Male mice	3 months	/	HF(LAD)		LVESD,LVEDD↑
Yang, T 2020 [27]	Male rat	240g	/	HF(LAD)	10 days; 60days	LVEF↓, LVFS↓

Abbreviations: **HF** = heart failure; **LAD** = ligating the LAD coronary artery; **TAC** = Transverse aortic constriction; **LV** = left ventricle; **FS** = fractional shortening; **LVESD** = Left Ventricular End Systolic Diameter; **LVEDD** = left ventricular end-diastolic diameter; **BNP** = Brain Natriuretic Peptide; **ESD** = end-systolic diameter; **EDD** = end-diastolic diameter; **ESA** = end-systolic area; **EDA** = end-diastolic area; **LVIDd** = left ventricular internal dimension-diastole; **LVIDs** = left ventricular internal dimension in systole; **EDV** = end-diastolic volume; **ESV** = end-systolic volume; **IVSd** = interventricular septal defect; **IVSs** = interventricular septal systole; **LVFS** = left ventricular fractional shortening; **MABP** = mean arterial blood pressure; **LVDd** = left ventricular diastolic dimension; **PWTd** = posterior wall thickness in diastole.

### 2. Qualitative analysis

The results of using NOS to evaluate the risk of bias and the method quality of 16 clinical studies were shown in Table 3. The mean score of NOS in the included clinical studies was 7.3 with the total scores ranging from 75% - 100%. One included clinical study was assessed by Cochrane Risk of Bias Tool and was found to be at low risk of bias (Table 4). According to the assessment of SYRCLE’s ROB tool, the included experimental studies displayed unclear of their sequence generation, random outcome assessment, blinding methods and other sources of bias. Most studies were low risk in baseline characteristics, random housing, incomplete outcome data, and selective outcome reporting. More details of the risk of bias of experimental studies were in Table 5.

**Table 3:** Newcastle–Ottawa Scale (NOS) of included clinical studies.

Author	Year	Method	Sample Size	Score
Roy, B [18]	2017	case-control study	48	8
Mueller, K [33]	2020	case-control study	137	7
T AN I A C. E S [16]	2006	cross-sectional study	52	7
Suzuki, H [9]	2016	case-control study	80	6
Frey, A [15]	2021	cohort study	148	8
Suzuki, H [21]	2020	cohort study	70	7
Niizeki, T [29]	2019	prospective study	491	8
Woo, M. A [30]	2015	case-control study	51	7
Yun, M [37]	2020	case-control study	117	6
Woo, M. A [32]	2005	case-control study	22	7
Wykrętowicz, A [31]	2019	case-control study	63	6
Menteer, J [34]	2010	case–control study	14	7
Woo, M. A [35]	2009	case-control study	62	8
Almeida, O. P [17]	2012	case-control study	155	7
Park, B [22]	2016	case-control study	80	6
Ichijo, Y. [19]	2020	cross-sectional study	63	7

**Table 4:** Cochrane Risk of Bias Tool of included clinical studies.

Author, year	Random Sequence Generation	Allocation Concealment	Blinding of Participants & Personnel	Blinding of Outcome Assessment	Incomplete Outcome Data	Selecting Reporting	Other Sources of Bias
Pan, A, 2013 [20]	low risk	low risk	low risk	low risk	low Risk	high Risk	high Risk

**Table 5:** SYRCLE’s risk of bias tool of animal experiments.

Author, year	Sequence generation (Selection bias)	Baseline characteristics (Selection bias)	Allocation concealment (Selection bias)	Random housing (Performance bias)	Blinding (Performance bias)	Random outcome assessment (Detection bias)	Blinding (Detection bias)	Incomplete outcome data (Attrition bias)	Selective outcome reporting (Reporting bias)	Other sources of bias
Shinoda Y, 2016 [38]	unclear	unclear	unclear	low risk	unclear	unclear	unclear	low risk	high risk	unclear
Lijun zhang, 2019 [39]	unclear	low risk	unclear	low risk	unclear	unclear	unclear	low risk	low risk	unclear
C.Liu, 2014 [14]	unclear	low risk	unclear	low risk	unclear	unclear	unclear	high risk	low risk	unclear
Frey, A 2014 [24]	unclear	low risk	high risk	low risk	unclear	unclear	unclear	low risk	low risk	unclear
Koji Ito, 2013 [66]	unclear	unclear	unclear	unclear	unclear	unclear	unclear	unclear	high risk	unclear
Y. Zhou, 2020 [40]	unclear	unclear	high risk	low risk	unclear	unclear	unclear	unclear	low risk	unclear
Yingbin Ge, 2020 [41]	unclear	low risk	unclear	unclear	unclear	unclear	unclear	low risk	low risk	unclear
Austin T. H. Duong, 2019 [25]	unclear	low risk	unclear	low risk	unclear	unclear	unclear	unclear	low risk	unclear
Camilo Toledo, 2019 [10]	unclear	unclear	unclear	low risk	unclear	unclear	unclear	unclear	low risk	unclear
H. Suzuki, 2015 [36]	unclear	unclear	high risk	low risk	unclear	unclear	unclear	low risk	low risk	unclear
Kim, Lee, Kim, 2017 [26]	unclear	low risk	unclear	low risk	unclear	unclear	unclear	unclear	low risk	unclear
Md Rezaul Islam. 2021 [28]	unclear	low risk	unclear	low risk	unclear	unclear	unclear	low risk	high risk	unclear
Yang, T 2020 [27]	unclear	low risk	low risk	low risk	unclear	unclear	unclear	low risk	high risk	unclear

### 3. Obvious cognitive dysfunction and depression in HF

In most of the clinical researches, the measurements used to evaluate cognitive function in HF patients varied, including MMSE, MoCA, TMT-A, TMT-B, GDS, BDI-II, WMS-R, HDS-R, CAMCOG, CVLT tests (more details shown in Table 6). Three studies reported the attention or executive function deficit [15–17] in HF patients and four studies presented memory impairment [9, 16, 18, 19]. Moreover, one study particularly described the poor abilities of language, remote memory, praxis, calculation, abstract reasoning, and perception subscales of HF patients [16]. While two studies believed there were no changes in cognition. In addition, several studies (*n = 4*) also proved the depressive symptoms in heart failure [9, 20–22]. However, there were six studies did not detect the cognitive function in HF patients.

**Table 6:** Characteristics of cognition and hippocampal changes in clinical studies.

Author, year	Cognitive test	Cognitive function	Method(s) for hippocampal change	Hippocampal damage	Hippocampal damage regions
Roy, B 2017 [18]	MoCA, Beck depression/ anxiety inventory (BDI/BAI)	HF group: significant **decrease** in MoCA, BDI scores and delayed recall compared with control group.	MRI	HF group: significant **decrease** in cerebral blood flow (CBF) compared with control group.	Right and left side
Mueller, K 2020 [33]	Neuropsychological tests battery	No differences in attention, executive function and memory between groups.	MRI	CAD with HF group: gray matter density **reduced** significantly compared with LIEF group.	N/A
Pan, A 2013 [20]	BDI-II	HF group: significant **increase** in BDI-II and TMT-B scores compared with control group.	MRI/Visual Assessment	HF group: significant difference showed in hippocampal atrophy compared with control group.	Right hippocampus
	TMT-B				
TAN I A C. T. F. A L V E S 2006 [16]	MMSE, CAMCOG	HF groups: significant **decrease** in MoCA and CAMCOG scores compared with healthy group.	^99m^Tc-SPECT	MDD-HF group: significant **decrease** in CBF compared with non-depressed HF group and healthy group.	Right posterior hippocampus/posterior para-hippocampal gyrus/Left anterior para-hippocampal gyrus/anterior hippocampus
Suzuki, H 2016 [9]	Psychological tests (MMSE,GDS,WMS-R,IM,DM)	Stage C HF patients: significant **increase** in GDS scores and **decrease** in IM and DM scores compared with control group.	MRI	Stage C HF patients: significant **decrease** in CBF compared with control group.	Posterior hippocampus postero-posterior hippocampus
Frey, A 2021 [15]	Psychological test battery	The intensity of attention **declined** in HF patients over 3years.	MRI	The mean hippocampal volume **declined** in HF patients over time.	Right side
		No differences in selectivity of attention and working memory.			
Suzuki, H 2020 [21]	Psychological tests (GDS, WMS-R)	The GDS scores **increased** in HF patients.	MRI	The anterior hippocampal blood flow was negatively correlated with changes in PWT in HF patients.	anterior and posterior hippocampal
Niizeki, T 2019 [29]	HDS-R	The patients with cardiac events (z-scores high group): significant **decrease** in HDS-R scores	MRI	The patients with cardiac events: significant **increased** prevalence of hippocampal atrophy compared with patients without cardiac events	N/A
Woo, M. A 2015 [30]	N/A	N/A	MRI	HF group: significant decline in right hippocampal volume.	Right hippocampus; CA1 and CA3 region
Yun, M 2020 [37]	N/A	N/A	18F-FDG PET/CT imaging	HF group: significant decrease in glucose metabolism of hippocampus	Right hippocampus
Woo, M. A 2005 [32]	N/A	N/A	MRI	HF group: significant decrease in gray matter of hippocampus	N/A
Wykrętowicz, A 2019 [31]	N/A	N/A	MRI	HF group: significant reduction of hippocampus volumes	N/A
Menteer, J 2010 [34]	N/A	N/A	MRI	HF group: significant gray matter loss in hippocampus	Right mid-hippocampus
Woo, M. A 2009 [35]	N/A	N/A	MRI	HF group: significant higher T2 relaxation values(loss of gray and white matter) in hippocampus.	Right hippocampus

Abbreviations: **Psychological test battery** include TAP, VVM2, WMSR, RET, H5PT tests(**TAP** = test battery of attentional processes; **VVM2** = the Visual and Verbal Memory Test; **WMS-R** = the Wechsler Memory Scale revised; **RET** = the Regensburger Word Fluency Test; **H5PT** = hamasch 5-point test revised). **Neuropsychological tests battery** include TMT-A, TAP,TMT-B, CVLT, ROCF (**TMT-A** = trail making test A; **TAP** = test battery of attentional processes; **TMT-B** = trail making test B; **RWT** = Regensburg word fluency test; **CVLT** = California verbal learning test; **ROCF** = Rey-Osterrieth complex figure test). **MoCA** = Montreal Cognitive Assessment test. **BDI-II** = the Beck depression inventory. **BAI** = the Beck anxiety inventory. **MMSE** = Mini-Mental State Examination. **CAMCOG** = Cambridge Mental Disorders of the Elderly Examination. **GDS** = Geriatric Depression Scale. **WMS-R** = the Wechsler Memory Scale-revised. **IM** = immediate memory. **DM** = a test of delayed memory. **PWT** = Posterior wall thickness; **HDS-R** = the Revised Hasegawa’s Dementia Scale; BA27/30, the right posterior hippocampus and posterior para-hippocampal gyrus; BA28/34/35/36, left anterior para-hippocampal gyrus. **GMD** = gray matter density. **z-scores** = refers to the measured value of a growth index of the tested population and reference to the average value of this index and the ratio of the overall standard deviation of this index. The z-score in patients with cardiac events was significantly higher than that in patients without cardiac events.

In animal studies, global cognition of animals was evaluated by passive avoidance tasks, active avoidance tasks, tail suspension test, forced swim test, sucrose preference test, open field tests, long-term potentiation recording, elevated plus maze, Y-maze test, object-in-place memory task, object oddity perceptual task and Morris water maze task*(n = 13)* (more details shown in Table 7). The reduction of learning [23] and memory in HF animals existed in six studies, and memory impairment manifested in short-term recognition memory [24], OiP memory [25], spatial memory [10, 26, 27], hippocampus-dependent memory [28].

**Table 7:** Characteristics of cognitive function and hippocampal and frontal lobe changes of HF rats in included experimental studies.

Author, year	Behavior tests	Cognitive function	Changes of hippocampus	Mechanisms or intervention
Shinoda Y, 2016 [38]	TST; SPT forced swim test;	Depression-like behaviors	Expression of σ1-receptor in the CA1 region and dentate gyrus↓	Increases in plasma corticosterone (CORT) levels; Corticosteroids
Lijun zhang, 2019 [39]	SPT, OFT	Depressive behaviors	Expression of 5-HT, 5-HT receptor↑	Ginkgo biloba Extract
C. Liu, 2014 [14]	LTP	Suppression of long-term potentiation	Levels of MDA and H2O2↑; Cu/Zn-SOD activity↓; NR2B expression↓	Activation of p-Akt/Akt
Frey, 2014 [24]	SPT,EPM, LDB, OFT; OR	Depression and anhedonia; reduced exploratory behavior; deficits in stress-coping; less habituation to new environment; deficits in short-term recognition memory	Transcriptional up-regulation of HIF-3α; retinoid-related orphan receptor-alpha↑; gene expression of Kif5b and Gabrb2↓	Serotonin system; differences of RNA expression of several hippocampal regions
Koji Ito, 2013 [66]	TST; Y-maze test	Decreased spontaneous alternation	Expression of sigma-1 receptor↓	Sigma-1 receptor
Y. Zhou, 2020 [40]	EPM, LDB, OFT	Exhibiting anxiety-like behavior	KDM6B↓; SIRT1↑; IL-1β, Bax, cleaved-caspase 3 proteins↑	MI-induced neuro-inflammation and neuronal apoptosis; downregulation of KDM6B but upregulation of SIRT1 signaling
Yingbin Ge 2020 [41]	OFT, SPT	Depression-like behaviors	Expression of 5-HT↑; IL-1β↑	Ginkgolide B attenuates myocardial infarction-induced depression-like behaviors
Austin T. H. Duong, 2019 [25]	OiP, Object oddity perceptual task	Impairment in OiP memory; object oddity discrimination in HF remains intact	Basal dendrite length increases↑; differences predominantly mapped to metabolic pathways	Core circadian mechanism
Camilo Toledo, 2019 [10]	MWM	Learning and memory impairment; spatial memory deficits	Expression levels of active β-catenin, pGSK-3β↓	Wnt/β-catenin signaling↓
H. Suzuki, 2015 [36]	24-hour LAM	Depressive symptoms	Decrease in gray matter concentration, neurogenesis and neurite outgrowth; increase in the number of astrocytes	--
Kim, Lee, 2017 [26]	Morris water maze task	Impairment of spatial memory	Cell death in the area of the hippocampus	Ang II receptor mediated cell death.
Md Rezaul Islam, 2021 [28]	OFT	Impaired hippocampus-dependent memory consolidation	Down-regulation of hippocampal genes	Reduced neuronal H3K4 methylation
Yang, T 2020 [27]	Morris water maze task	Impairment of spatial memory	Brain glucose metabolism of frontal cortex significantly lower in 60 days HF rats and higher in 10 days HF rats.	/

Abbreviations: **OFT** = open field tests; **SPT** = Sucrose preference test; **TST** = Tail suspension test; **LDB** = light dark box; **EPM** = elevated plus maze; **OiP** = Object-in-place memory task; **LTP** = Long-term potentiation recording; **EPM** = elevated plus maze; **OR** = object recognition; **MWM** = Morris Water Maze; **LAM** = locomotor activity measurement.

### 4. Abnormalities of hippocampus in HF patients and animal models

#### 4.1 Differences in hippocampal volume of HF patients

Five clinical studies (5/17) confirmed that the mean hippocampal volume decreased in HF patients compared to control groups [15, 20, 29–31]. Among these studies, three of them found significant hippocampal atrophy mainly reflected in the right hippocampus [15, 20, 30]. There were no studies about hippocampal volume in animal experiments. The detailed results are shown in Table 6.

#### 4.2 CBF alternations of the hippocampus in HF patients

As illustrated in Table 6, several studies(*n = 4*) indicated hippocampus showed lower CBF values in HF patients [9, 16, 18, 21]. However, in one study, CBF in the whole hippocampus of HF patients tended to be lower without statistical significance (*P = 0*.*279*), while CBF had a regional significant reduction, mainly reflected in the most posterior portion of the hippocampus [9]. Obviously, CBF decrease was also observed in anterior hippocampal gyrus of some studies(*n = 2*) [16, 21]. There were no studies about hippocampal CBF in animal experiments.

#### 4.3 Gray matter decrease in hippocampus in HF patients

Other hippocampal injuries in HF were reduction of gray matter. Four studies [32–35] showed a significant GMD decrease in hippocampus and surprisingly, almost all of them reported this change in the right side of hippocampus except one study did not mention regional change. Similarly, there was one experimental study also observed a decrease in gray matter concentration in HF rats hippocampus [36]. The detailed results are shown in Table 6.

#### 4.4 Decrease in glucose metabolism of hippocampus in HF patients

As illustrated in Table 6, one study [37] reported the significant decrease in glucose metabolism of hippocampus. Interestingly, this study also represented that this kind of changes exhibited in the right side of hippocampus.

#### 4.5 Hippocampal damages in HF animal model

Clinical research about hippocampal damages in heart failure is just limited to phenomenological changes, and deeper potential molecular mechanisms should be explored through basic experimental researches. Among experimental studies, each study analyzed different genes or proteins with statistical significance. The detailed results are shown in Table 7.

### 5. Abnormalities of frontal lobe in HF patients and animal models

There were four clinical studies and one experimental study involved the changes of frontal lobe in heart failure. The detailed results are shown in Tables 7 and 8. Two studies also reported the loss of gray matter of frontal lobe in HF [17, 33]. The other two studies showed significant decreased functional connectivity and mean oxyhemoglobin concentrations in frontal gyrus, respectively [19, 22].

**Table 8:** Characteristics of cognition and changes of frontal lobe in clinical studies.

Author, year	Cognitive test	Cognitive function	Method(s) for frontal lobe change	frontal lobe damage	frontal lobe damage regions
Almeida, O. P 2012 [17]	CAMCOG, CVLT, digit coding/copying	HF group: significant **decrease** in CVLT and digit coding scores compared with control group.	MRI	HF group: significant loss of gray matter in frontal gyrus compared with control group.	Right inferior/middle/ precentral frontal gyrus; left middle frontal gyri
Park, B 2016 [22]	MoCA, BDI, BAI	HF group: significant **decrease** in MoCA, BDI, BAI scores compared with control group.	MRI, brain network analysis	HF group: significant decreased functional connectivity in frontal gyrus.	N/A
Ichijo, Y. 2020 [19]	VFT, CES-D, STAI, MMSE	HF group: significant **increase** in VFT, STAI and MMSE scores compared with control group.	Near-Infrared Spectroscopy	HF group: significant lower mean oxyhemoglobin concentrations of frontal region compared with control group.	N/A
Mueller, K 2020 [33]	Neuropsychological tests battery	No differences in attention, executive function and memory between groups.	MRI	CAD with HF group: gray matter density **reduced** significantly in whole frontal cortex compared with LIEF group.	N/A

Abbreviations: **CAMCOG** = Cambridge Mental Disorders of the Elderly Examination. **CVLT** = California verbal learning test; **MoCA** = Montreal Cognitive Assessment test. **BDI-II** = the Beck depression inventory. **BAI** = the Beck anxiety inventory. **MMSE** = Mini-Mental State Examination. **VFT** = verbal fluency task; **CES-D** = Center for Epidemiologic Studies Depression Scale. **STAI** = State-Trait Anxiety Inventory.

## Discussion

Heart failure patients shows obvious cognitive impairment or depression, which increases the mortality and rehospitalization rate of HF patients. The hippocampus and frontal lobe were considered to be the most important brain regions for cognitive flexibility (such as cognition and mood) during the HF stage. However, there are scarcely any systematic reviews about changes of the hippocampus and frontal lobe in HF patients and animal models.

### 1. Statement of key findings

This systematic review synthesized available studies about changes of hippocampus and frontal lobe in HF and finally included 30 studies (17 clinical researches and 13 animal studies). This review scientifically summarized the cognitive dysfunction and depressive symptoms in HF which reflected in poor abilities of attention, executive function, learning and memory, and anhedonia, reduced exploratory or anxiety-like behavior in both HF patients and animals. Importantly, this review integrally concluded changes of the hippocampus and frontal lobe in HF patients, and these changes mainly showed in volume atrophy, decreased CBF, reduced gray matter and glucose metabolism. Moreover, hippocampal damages of HF patients were mainly observed in the right side. According to the animal studies, the results showed the inflammation, synaptic abnormalities, and neurotransmitter disorders of hippocampus and frontal lobes in HF animal model, which complemented the clinical observations by providing potential mechanistic explanations of the changes in the hippocampus and frontal lobes. To our knowledge, this is the first systematic review to focus on the hippocampal and frontal damages in HF patients and animal model.

### 2. Obvious cognitive impairment and depression exhibited in HF

In this systematic review, we found out heart failure may be considered as an important reason for cognitive dysfunction and depression. Several studies included in our results represented that there was not an obvious relationship between cognitive impairment caused by HF and comorbidities (such as diabetes and CAD) [15, 29, 33]. Moreover, the experimental studies also demonstrated similar results. In the HF model without any confounding factors, animals all showed obvious cognitive impairment. These results suggested that heart failure may be one of the factors leading to cognitive impairment. However, there were two clinical studies that showed paradoxical results that there was no difference in attention, working memory, or executive function [15, 33]. The most principal reason was the differences in the range and specificity of the instruments used to assess cognition. The MoCA or MMSE was the instrument most studies were willing to preferring. For another, the heart failure severity of patients in these two studies was lighter, which may influence the cognitive results. Coincidentally, in our systematic review, we also found out the presence of cognitive impairment in an experimental model of HFpEF [10]. However, according to our searching results, we cannot judge the difference in cognitive impairment between the HFpEF and HFrEF models. Finally, most of our studies [10, 28] suggested that decreased cognitive performance observed in HF rats may dependent on hippocampally-dependent mechanisms. Depressive symptoms were also found to be prevalent in HF patients in this systematic review [9, 18, 20]. Similarly to clinical studies, depression-like behaviors were also found in HF animals in most of the experimental studies(*n = 6*) [24, 36, 38–41]. The previous study represented that frontal dysfunction has been observed in patients with depression, which may be further associated with cognitive impairment [42].

Moreover, cognitive impairment and depression in HF may be related to brain-derived neurotrophic factor (BDNF). BDNF plays a crucial role in memory formation and the hippocampus contains a high concentration of it [43]. Some studies reported that lower blood BDNF levels were associated with a higher incidence of dementia and depression in CHF patients [44, 45]. While since BDNF could be produced in the skeletal muscle, another research suggested that the decrease in serum BDNF levels may be due to the physical inactivity in HF patients [46]. Therefore, we believed that further studies are needed to investigate BDNF levels in exosomes derived from neurons, rather than plasma in HF patients, which could better-reflected abnormalities in the brain.

### 3. Hippocampal and frontal damage alterations---and possible mechanism

#### 3.1 Reduction in CBF

In this review, the results represented that the CBF of the hippocampus decreased in HF for regional significant reduction not only in the posterior hippocampus but also in the anterior hippocampal gyrus. The hippocampus has different functions and neuroanatomy in the different subregions of the anterior-posterior axis [47]. The posterior hippocampus plays a major role in cognitive functions and memory, while the anterior hippocampus performs emotion and stress. The hippocampus is a very vascular structure and susceptive to changes in blood flow and hypoxemia [48]. It has been reported that reduced CBF in the hippocampus may possibly associate with poorer cognitive function and depressive symptoms in AD [49].

Some potential pathophysiology mechanisms may contribute to decreased regional CBF in HF. The possible mechanism for brain damage in HF patients may be due to cardiac dysfunction and decreasing cardiac index. The reduction of low cardiac output is may lead to ischemia which affects both vasculature and endothelial function. Altered endothelial function can result in abnormal cerebral autoregulation, which is mainly reflected in decreased CBF in HF patients. Cerebral autoregulation maintains a stable CBF over a wide range of mean arterial pressure and can be affected by the renin-angiotensin system [50], which is a central neurohormonal response to control cardiovascular and renal function. Therefore, B-type natriuretic peptide (BNP) neurohormones, which is released as a response to increased ventricular wall stretch and marker of the severity of HF [51], may cause a reduction of CBF. The high levels of BNP may result in a more active neurohormonal system in HF patients, and this may cause the distortion of brain autoregulation [51]. Thus, the hippocampus might be especially prone to damage caused initially by a disruption of blood flow. In addition, central venous pressure (CVP) represents the pressure of the thoracic vena cava near the right atrium, and patients with HF often accompany increased CVP [52]. While an increase in CVP can affect brain oxygen and capacity, causing a decrease in oxygen saturation and affecting cognitive function. Of note, one study represents that the influence of CVP on cerebral perfusion was most pronounced in low arterial flow states [53]. Moreover, variable degrees of carotid obstruction in HF might have contributed to the CBF reductions [54]. The abnormal hippocampal CBF in CHF patients may be reversible. Furthermore, one other possibility is that reduction of CBF in HF patients may be influenced by cerebrovascular reactivity. The previous study evaluated CO_2_-reactivity and blood flow velocities of the middle cerebral arteries in CHF patients and demonstrated the impairment of cerebrovascular reactivity [55]. Lower CO_2_ contributes to reduced cerebral arteriolar dilatation, which leads to reduced CBF [56]. And the decrease in cerebrovascular reactivity had a relationship with the decline in cardiac function.

#### 3.2 Reduction in volume

The results in our systematic review demonstrated that the right hippocampus was significantly atrophied in HF patients. Previous researches have indicated that the right hippocampal functionality may be impaired in depression [57, 58]. The reason why brain atrophy occurred in the right hippocampus not the left side may be that the hippocampus exhibits lateralization [59]. This trend may also reflect preferential right-sided activation for sympathetic regulation, thus being more metabolically demanding of perfusion, which may be unmet by compromised cerebral autoregulation in HF. It was also consistent with our searching results of changes of CBF in hippocampus of HF patients.

#### 3.3 Decreased gray matter density and glucose metabolism

In this systematic review, the gray matter density and glucose metabolism obviously decreased in the right hippocampus in HF, and one study demonstrated this regional GM was positively correlated with EF and negatively with NT-proBNP [33]. On the other hand, gray matter density was also reduced in the frontal lobes of HF patients, and glucose metabolism of chronic HF rats also declined in the frontal lobes. It has been shown that vascular dementia (VD) patients had greater deficits in frontal–executive functions, verbal fluency, attention, and motor function when compared to Alzheimer’s disease (AD) of similar severity [60]. Therefore, we believe that cognitive impairment in patients with HF may be similar to vascular dementia to some extent.

Several previous studies suggested that the brain gray matter density was related to cardiovascular impairment [17, 61]. Gray matter, an important part of the central nervous system, was consisted of a large number of neuronal bodies and their dendrites. As in our previous study [27], we also found out that the number of neurons and synapses significantly decreased and Nissl bodies disappeared in HF rats. In other words, our previous studies indirectly proved the decreased gray matter density of HF patients in clinical.

These decreases in volume, gray matter density, and glucose metabolism of the hippocampus and frontal lobes may be related to low cardiac output and cerebral hypoxia. As discussed earlier in this review, low cardiac output in HF patients results in ischemia, which affects cerebral autoregulation and reduces cerebral blood flow. Subsequently, this decrease in CBF can contribute to a hypoxic environment and a shortage of glucose, leading to neuronal and glial necrosis in the hippocampus or frontal lobes [62]. It is likely that neuronal degeneration in a cerebral hypoxic environment is a slower process and may be due to a combination of excitotoxicity, producing necrosis, and apoptosis. In addition, the decrease in the mean oxyhemoglobin concentration in the frontal region may also contribute to brain structural abnormalities.

#### 3.4 Potential pathological mechanisms of hippocampal and frontal damages in HF animal model the exact mechanism

This systematic review generalizes the molecular processes and gene expression of the hippocampus in the HF model. Firstly, that HF may induce neuro-inflammation and cell apoptosis in the hippocampus. Several studies in our results demonstrated that the levels of IL-1β, Bax, cleaved-caspase 3 proteins increased in the hippocampus of HF rats [40, 41, 63], which may have a relationship with Ang II receptor and upregulation of SIRT1. Meanwhile, we also found two studies that suggested the expression of the sigma-1 receptor in the hippocampus was reduced in the heart failure model. Brain sigma-1 receptor(σ1R) plays a major role in inflammation, neurite outgrowth or Ca2+ signal regulation, and is reported as the key molecule of the pathogenesis in depression or cognitive impairment [64]. Secondly, HF animals were used to cause the alternations of neurotransmitters and their receptors in the hippocampus. As shown in our results, the levels of 5-HT increased in the hippocampus in HF rats in our results, which contributed to the development of depression. Moreover, the expression of NR2B protein, a major functional component of the hippocampal NMDA receptors, was obviously reduced in the MI model group. Thirdly, molecular mechanisms involving synaptic changes also were observed in HF model. One included experimental study indicated the reduction of the Wnt signaling pathway in the hippocampus. Wnt signaling pathway has been largely implicated in the regulation of synaptic assembly, neurotransmission and synaptic plasticity in the adult nervous system [65]. Lastly, from the gene expression, downregulation in gene expression of Kif5b and Gabrb2 were observed in HF, which were responsible for axonal vesicular transport, and regulation of synaptic transmission, respectively. Our results suggested that hippocampal gene expression changes were observed in mice suffering from HF. One of the included experimental studies reported the decreased levels of the euchromatin mark H3K4me3 in the hippocampus. While the proper neuronal H3K4me3 is essential for memory consolidation in previous research.

### 4. Limitations

A major limitation of the included clinical studies reviewed is that the potential confounders for cognitive dysfunction could influence the results of these studies, including education level, without dividing into groups according to HFpEF or HFrEF, and diabetes. The other limitation is that the feeding time after MI surgery is included experimental studies is various and ranged from 6 hours to 16 weeks, which may have an influence on the results of cognition and hippocampal changes in the HF model. Finally, this review only focused on hippocampus while other putative brain structures that should be involved in future research, such as frontal lobes, amygdala, locus coeruleus, hypothalamus.

### 5. Implications for future researches

Cognitive impairment or depression in HF patients will induce the poor ability of self-care and poor quality of life in patients, while the hippocampal damages may provide a structural and functional explanation for the disabilities in cognition and depression. These indicated that the heart and brain are interconnected and interact with each other pathologically. And unfortunately, few studies are focusing on the effect of therapeutic interventions specifically for HF on cognitive function. This systematic review identifies underlying mechanisms of the hippocampus for cognitive disorders or depression in HF, which may provide treatment strategies and targets for intervention. In addition, it also could present data for future basic researches and further strengthen the notion that brain and heart function are tightly linked.

## Conclusions

**Fig 2:**
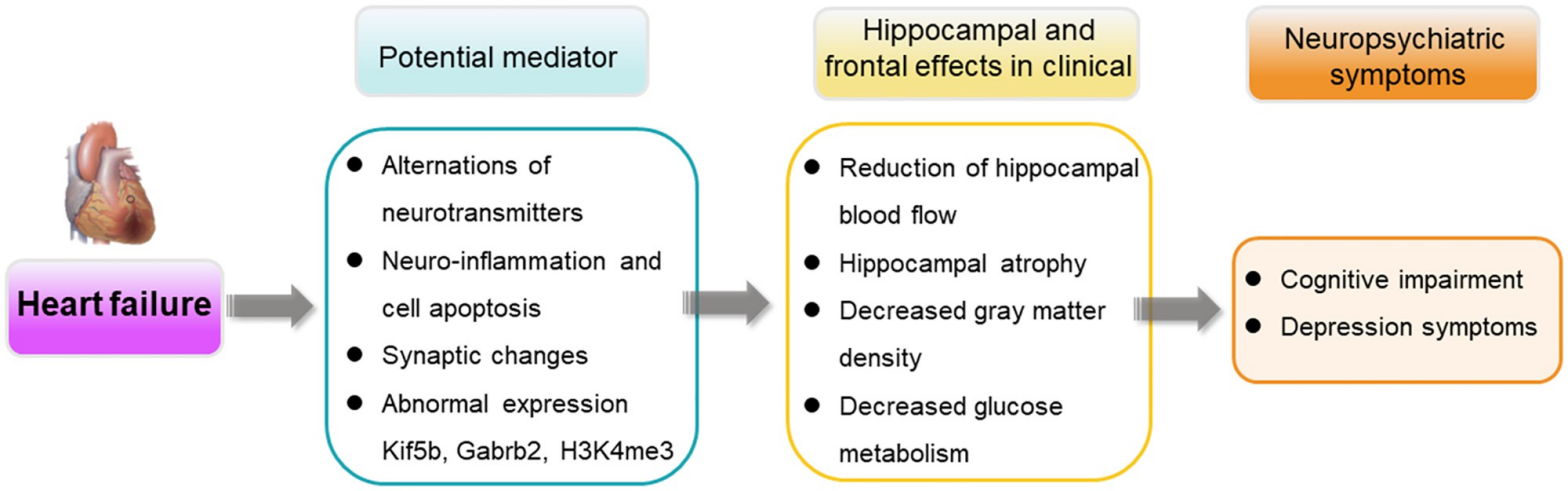
Graphical abstract.

A total of 30 literature studies were included to review the hippocampal and frontal changes in HF patients and animal models. Cognitive dysfunction in HF patients and animal models mainly showed poor abilities of attention, executive function, learning and memory, and anhedonia reduced exploratory or anxiety-like behavior. Hippocampal injuries of HF patients were shown in the reduction of volume, CBF, glucose metabolism, and gray matter, which were mainly observed in the right hippocampus. The frontal damages of HF patients were only in reduced gray matter and have no differences between the right and left sides. The included HF animal model studies demonstrated the inflammation and apoptosis, synaptic reduction, and neurotransmitter disorders of the hippocampus and frontal lobes, which contributed to the loss of gray matter and volume. The graphical abstract was shown in Fig 2. The results of HF animal model studies complemented the clinical observations by providing potential mechanistic explanations of the changes in the hippocampus and frontal lobes. Finally, this systematic review provided data and therapeutic targets for the prevention and treatment of cognitive impairment and depression after heart failure.

## Supporting information

S1 Checklist PRISMA 2020 checklist. (DOCX)

S1 File (DOCX)

## Abbreviations

AD: Alzheimer’s disease
BAI: the Beck anxiety inventory
BDI-II: the Beck depression inventory
BNP: brain natriuretic peptide
CAMCOG: Cambridge mental disorders of the elderly examination
CBF: cerebral blood flow
CES-D: center for epidemiologic studies depression scale
CI: cognition impairment
CVLT: California verbal learning test
DM: a test of delayed memory
EDA: end-diastolic area
EDD: end-diastolic diameter
EDV: end-diastolic volume
ESA: end-systolic area
ESD: end-systolic diameter
ESV: end-systolic volume
FS: fractional shortening
GDS: Geriatric Depression Scale
GMD: gray matter density
H5PT: hamasch 5-point test revised
HDS-R: the revised Hasegawa’s dementia scale
HF: heart failure
HFpEF: heart failure with preserved ejection fraction
HFrEF: heart failure with reduced ejection fraction
IM: immediate memory
LAD: left anterior descending coronary
LVDd: left ventricular diastolic dimension
LVEDD: left ventricular end-diastolic diameter
LVEF: left ventricular ejection fraction
LVESD: left ventricular end systolic diameter
MABP: mean arterial blood pressure
MCI: mild cognitive impairment
MI: myocardial infarction
MMSE: Mini-Mental State Examination
MoCA: Montreal Cognitive Assessment test
NT-proBNP: N-terminal prohormone of brain natriuretic peptide
NYHA: New York Heart Association
PWTd: posterior wall thickness in diastole
RET: the Regensburger Word Fluency Test
ROCF: Rey-Osterrieth complex figure test
RWT: Regensburg word fluency test
STAI: State-Trait Anxiety Inventory
TAP: test battery of attentional processes
TEM: Transmission Electron Microscopy
TMT-A: trail making test A
TMT-B: trail making test B
VFT: verbal fluency task
VVM2: the visual and verbal memory test
WMS-R: the Wechsler Memory Scale revised

## References

[1] Ziaeian B and Fonarow GC (2016) Epidemiology and aetiology of heart failure. Nat Rev Cardiol 13:368–78. doi: 10.1038/nrcardio.2016.25 26935038 PMC4868779

[2] Rutledge T, Reis VA, Linke SE, Greenberg BH and Mills PJ (2006) Depression in heart failure a meta-analytic review of prevalence, intervention effects, and associations with clinical outcomes. J Am Coll Cardiol 48:1527–37. doi: 10.1016/j.jacc.2006.06.055 17045884

[3] Cannon JA, Moffitt P, Perez-Moreno AC, Walters MR, Broomfield NM, McMurray JJV et al. (2017) Cognitive Impairment and Heart Failure: Systematic Review and Meta-Analysis. J Card Fail 23:464–475. doi: 10.1016/j.cardfail.2017.04.007 28433667

[4] Sbolli M, Fiuzat M, Cani D and O’Connor CM (2020) Depression and heart failure: the lonely comorbidity. Eur J Heart Fail 22:2007–2017. doi: 10.1002/ejhf.1865 32468714

[5] Ampadu J and Morley JE (2015) Heart failure and cognitive dysfunction. Int J Cardiol 178:12–23. doi: 10.1016/j.ijcard.2014.10.087 25464210

[6] Popp S, Frey A, Siren AL, Schmitt A, Hommers L, Ertl G, et al. (2014) Chronic heart failure and depression: anhedonia, motivation deficits, and functional brain changes in mice with myocardial infarction. European Neuropsychopharmacology 24:S294–S295.

[7] Ogren JA, Fonarow GC and Woo MA (2014) Cerebral impairment in heart failure. Curr Heart Fail Rep 11:321–9. doi: 10.1007/s11897-014-0211-y 25001614

[8] Havakuk O, King KS, Grazette L, Yoon AJ, Fong M, Bregman N, et al. (2017) Heart Failure-Induced Brain Injury. J Am Coll Cardiol 69:1609–1616. doi: 10.1016/j.jacc.2017.01.022 28335844

[9] Suzuki H, Matsumoto Y, Ota H, Sugimura K, Takahashi J, Ito K, et al. (2016) Hippocampal Blood Flow Abnormality Associated With Depressive Symptoms and Cognitive Impairment in Patients With Chronic Heart Failure. Circ J 80:1773–80. doi: 10.1253/circj.CJ-16-0367 27295999

[10] Toledo C, Lucero C, Andrade DC, Díaz HS, Schwarz KG, Pereyra KV, et al. (2019) Cognitive impairment in heart failure is associated with altered Wnt signaling in the hippocampus. Aging (Albany NY) 11:5924–5942. doi: 10.18632/aging.102150 31447429 PMC6738419

[11] Rosch KS and Mostofsky S (2019) Development of the frontal lobe. Handb Clin Neurol 163:351–367. doi: 10.1016/B978-0-12-804281-6.00019-7 31590741

[12] Hooijmans CR, Rovers MM, de Vries RB, Leenaars M, Ritskes-Hoitinga M and Langendam MW (2014) SYRCLE’s risk of bias tool for animal studies. BMC Med Res Methodol 14:43. doi: 10.1186/1471-2288-14-43 24667063 PMC4230647

[13] Heidenreich PA, Bozkurt B, Aguilar D, Allen LA, Byun JJ, Colvin MM, et al. (2022) 2022 AHA/ACC/HFSA Guideline for the Management of Heart Failure: Executive Summary: A Report of the American College of Cardiology/American Heart Association Joint Committee on Clinical Practice Guidelines. J Am Coll Cardiol 79:1757–1780. doi: 10.1016/j.jacc.2021.12.011 35379504

[14] Liu CH, Liu Y and Yang Z (2014) Myocardial infarction induces cognitive impairment by increasing the production of hydrogen peroxide in adult rat hippocampus. Neuroscience Letters 560:112–116. doi: 10.1016/j.neulet.2013.12.027 24361546

[15] Frey A, Homola GA, Henneges C, Mühlbauer L, Sell R, Kraft P, et al. (2021) Temporal changes in total and hippocampal brain volume and cognitive function in patients with chronic heart failure-the COGNITION.MATTERS-HF cohort study. European heart journal. doi: 10.1093/eurheartj/ehab003 33496311 PMC8060058

[16] Alves TC, Rays J, Fráguas R, Wajngarten M, Telles RM, Duran FL, et al. (2006) Association between major depressive symptoms in heart failure and impaired regional cerebral blood flow in the medial temporal region: a study using 99m Tc-HMPAO single photon emission computerized tomography (SPECT). Psychol Med 36:597–608. doi: 10.1017/S0033291706007148 16515732

[17] Almeida OP, Garrido GJ, Beer C, Lautenschlager NT, Arnolda L and Flicker L (2012) Cognitive and brain changes associated with ischaemic heart disease and heart failure. Eur Heart J 33:1769–76. doi: 10.1093/eurheartj/ehr467 22296945

[18] Roy B, Woo MA, Wang DJJ, Fonarow GC, Harper RM and Kumar R (2017) Reduced regional cerebral blood flow in patients with heart failure. European Journal of Heart Failure 19:1294–1302. doi: 10.1002/ejhf.874 28560737 PMC5633487

[19] Ichijo Y, Kono S, Yoshihisa A, Misaka T, Kaneshiro T, Oikawa M, et al. (2020) Impaired Frontal Brain Activity in Patients With Heart Failure Assessed by Near-Infrared Spectroscopy. J Am Heart Assoc 9:e014564. doi: 10.1161/JAHA.119.014564 31973606 PMC7033895

[20] Pan A, Kumar R, Macey PM, Fonarow GC, Harper RM and Woo MA (2013) Visual assessment of brain magnetic resonance imaging detects injury to cognitive regulatory sites in patients with heart failure. J Card Fail 19:94–100. doi: 10.1016/j.cardfail.2012.12.001 23384634 PMC4249656

[21] Suzuki H, Matsumoto Y, Sugimura K, Takahashi J, Miyata S, Fukumoto Y, et al. (2020) Impacts of hippocampal blood flow on changes in left ventricular wall thickness in patients with chronic heart failure. International Journal of Cardiology 310:103–107. doi: 10.1016/j.ijcard.2020.01.019 31955976

[22] Park B, Roy B, Woo MA, Palomares JA, Fonarow GC, Harper RM et al. (2016) Lateralized Resting-State Functional Brain Network Organization Changes in Heart Failure. PLoS One 11:e0155894. doi: 10.1371/journal.pone.0155894 27203600 PMC4874547

[23] Ma JC, Duan MJ, Li KX, Biddyut D, Zhang S, Yan ML, et al. (2018) Knockdown of MicroRNA-1 in the Hippocampus Ameliorates Myocardial Infarction Induced Impairment of Long-Term Potentiation. Cellular Physiology and Biochemistry 50:1601–1616. doi: 10.1159/000494657 30359966

[24] Frey A, Popp S, Post A, Langer S, Lehmann M, Hofmann U, et al. (2014) Experimental heart failure causes depression-like behavior together with differential regulation of inflammatory and structural genes in the brain. Frontiers in Behavioral Neuroscience 8:1–13. doi: 10.3389/fnbeh.2014.00376 25400562 PMC4215623

[25] Duong ATH, Reitz CJ, Louth EL, Creighton SD, Rasouli M, Zwaiman A, et al. (2019) The Clock Mechanism Influences Neurobiology and Adaptations to Heart Failure in Clock(Δ19/Δ19) Mice With Implications for Circadian Medicine. Sci Rep 9:4994. doi: 10.1038/s41598-019-41469-7 30899044 PMC6428811

[26] Kim MS, Lee GH, Kim YM, Lee BW, Nam HY, Sim UC, et al. (2017) Angiotensin II Causes Apoptosis of Adult Hippocampal Neural Stem Cells and Memory Impairment Through the Action on AMPK-PGC1 alpha Signaling in Heart Failure. Stem Cells Translational Medicine 6:1491–1503. doi: 10.1002/sctm.16-0382 28244243 PMC5689768

[27] Yang T, Lu Z, Wang L, Zhao Y, Nie B, Xu Q, et al. (2020) Dynamic Changes in Brain Glucose Metabolism and Neuronal Structure in Rats with Heart Failure. Neuroscience 424:34–44. doi: 10.1016/j.neuroscience.2019.10.008 31704495

[28] Islam MR, Lbik D, Sakib MS, Hofmann RM, Berulava T, Mausbach MJ, et al. (2021) Epigenetic gene expression links heart failure to memory impairment. Embo Molecular Medicine 13:17. doi: 10.15252/emmm.201911900 33471428 PMC7933944

[29] Niizeki T, Iwayama T, Ikeno E and Watanabe M (2019) Prognostic Importance of Hippocampal Atrophy in Patients With Chronic Heart Failure. Am J Cardiol 123:1109–1113. doi: 10.1016/j.amjcard.2018.12.035 30678833

[30] Woo MA, Ogren JA, Abouzeid CM, Macey PM, Sairafian KG, Saharan PS, et al. (2015) Regional hippocampal damage in heart failure. Eur J Heart Fail 17:494–500. doi: 10.1002/ejhf.241 25704495 PMC4651448

[31] Wykrętowicz A, Wykrętowicz M, Katulska K, Schneider A, Piskorski J and Guzik P (2019) Brain volume loss in heart failure and its association with markers of left ventricular function. Polish Archives of Internal Medicine 129:426–429. doi: 10.20452/pamw.4488 30870401

[32] Woo MA, Macey PM, Keens PT, Kumar R, Fonarow GC, Hamilton MA et al. (2005) Functional abnormalities in brain areas that mediate autonomic nervous system control in advanced heart failure. Journal of Cardiac Failure 11:437–446. doi: 10.1016/j.cardfail.2005.02.003 16105635

[33] Mueller K, Thiel F, Beutner F, Teren A, Frisch S, Ballarini T, et al. (2020) Brain Damage With Heart Failure Cardiac Biomarker Alterations and Gray Matter Decline. Circulation Research 126:750–764. doi: 10.1161/CIRCRESAHA.119.315813 31969053 PMC7077969

[34] Menteer J, Macey PM, Woo MA, Panigrahy A and Harper RM (2010) Central Nervous System Changes in Pediatric Heart Failure: A Volumetric Study. Pediatric Cardiology 31:969–976. doi: 10.1007/s00246-010-9730-9 20521036 PMC2948160

[35] Woo MA, Kumar R, Macey PM, Fonarow GC and Harper RM (2009) Brain Injury in Autonomic, Emotional, and Cognitive Regulatory Areas in Patients With Heart Failure. Journal of Cardiac Failure 15:214–223. doi: 10.1016/j.cardfail.2008.10.020 19327623 PMC2730774

[36] Suzuki H, Sumiyoshi A, Matsumoto Y, Duffy BA, Yoshikawa T, Lythgoe MF, et al. (2015) Structural abnormality of the hippocampus associated with depressive symptoms in heart failure rats. Neuroimage 105:84–92. doi: 10.1016/j.neuroimage.2014.10.040 25462699

[37] Yun M, Nie B, Wen W, Zhu Z, Liu H, Nie S, et al. (2020) Assessment of cerebral glucose metabolism in patients with heart failure by 18F-FDG PET/CT imaging. Journal of Nuclear Cardiology. doi: 10.1007/s12350-020-02258-2 32691347

[38] Shinoda Y, Tagashira H, Bhuiyan MS, Hasegawa H, Kanai H, Zhang C, et al. (2016) Corticosteroids Mediate Heart Failure-Induced Depression through Reduced σ1-Receptor Expression. PLoS One 11:e0163992. doi: 10.1371/journal.pone.0163992 27741227 PMC5065174

[39] Zhang LJ, Liu JY, Ge YB and Liu MY (2019) Ginkgo biloba Extract Reduces Hippocampus Inflammatory Responses, Improves Cardiac Functions And Depressive Behaviors In A Heart Failure Mouse Model. Neuropsychiatric Disease and Treatment 15:3041–3050. doi: 10.2147/NDT.S229296 31754303 PMC6825506

[40] Zhou Y, Tian QY, Zheng CF, Yang JG, Fan JM and Shentu YP (2020) Myocardial infarction-induced anxiety-like behavior is associated with epigenetic alterations in the hippocampus of rat. Brain Research Bulletin 164:172–183. doi: 10.1016/j.brainresbull.2020.08.023 32871241

[41] Ge YB, Xu W, Zhang LJ and Liu MY (2020) Ginkgolide B attenuates myocardial infarction-induced depression-like behaviors via repressing IL-1 beta in central nervous system. International Immunopharmacology 85:8. doi: 10.1016/j.intimp.2020.106652 32521492

[42] Nelson BD, Kessel EM, Klein DN and Shankman SA (2018) Depression symptom dimensions and asymmetrical frontal cortical activity while anticipating reward. Psychophysiology 55. doi: 10.1111/psyp.12892 28555883 PMC5704985

[43] Erickson KI, Miller DL and Roecklein KA (2012) The aging hippocampus: interactions between exercise, depression, and BDNF. Neuroscientist 18:82–97. doi: 10.1177/1073858410397054 21531985 PMC3575139

[44] Weinstein G, Beiser AS, Choi SH, Preis SR, Chen TC, Vorgas D, et al. (2014) Serum brain-derived neurotrophic factor and the risk for dementia: the Framingham Heart Study. JAMA Neurol 71:55–61. doi: 10.1001/jamaneurol.2013.4781 24276217 PMC4056186

[45] Takashio S, Sugiyama S, Yamamuro M, Takahama H, Hayashi T, Sugano Y, et al. (2015) Significance of low plasma levels of brain-derived neurotrophic factor in patients with heart failure. Am J Cardiol 116:243–9. doi: 10.1016/j.amjcard.2015.04.018 25983281

[46] Fukushima A, Kinugawa S, Homma T, Masaki Y, Furihata T, Yokota T, et al. (2013) Decreased serum brain-derived neurotrophic factor levels are correlated with exercise intolerance in patients with heart failure. Int J Cardiol 168:e142–4. doi: 10.1016/j.ijcard.2013.08.073 24029660

[47] Fanselow MS and Dong HW (2010) Are the dorsal and ventral hippocampus functionally distinct structures? Neuron 65:7–19. doi: 10.1016/j.neuron.2009.11.031 20152109 PMC2822727

[48] Balu DT and Lucki I (2009) Adult hippocampal neurogenesis: regulation, functional implications, and contribution to disease pathology. Neurosci Biobehav Rev 33:232–52. doi: 10.1016/j.neubiorev.2008.08.007 18786562 PMC2671071

[49] Schuff N, Matsumoto S, Kmiecik J, Studholme C, Du A, Ezekiel F, et al. (2009) Cerebral blood flow in ischemic vascular dementia and Alzheimer’s disease, measured by arterial spin-labeling magnetic resonance imaging. Alzheimers Dement 5:454–62. doi: 10.1016/j.jalz.2009.04.1233 19896584 PMC2802181

[50] Wanless RB, Anand IS, Gurden J, Harris P and Poole-Wilson PA (1987) Regional blood flow and hemodynamics in the rabbit with adriamycin cardiomyopathy: effects of isosorbide dinitrate, dobutamine and captopril. J Pharmacol Exp Ther 243:1101–6. 3320344

[51] Choi BR, Kim JS, Yang YJ, Park KM, Lee CW, Kim YH, et al. (2006) Factors associated with decreased cerebral blood flow in congestive heart failure secondary to idiopathic dilated cardiomyopathy. Am J Cardiol 97:1365–9. doi: 10.1016/j.amjcard.2005.11.059 16635612

[52] Parkin WG and Leaning MS (2008) Therapeutic control of the circulation. J Clin Monit Comput 22:391–400. doi: 10.1007/s10877-008-9147-7 19002596

[53] Sakamoto T, Duebener LF, Laussen PC and Jonas RA (2004) Cerebral ischemia caused by obstructed superior vena cava cannula is detected by near-infrared spectroscopy. J Cardiothorac Vasc Anesth 18:293–303. doi: 10.1053/j.jvca.2004.03.008 15232808

[54] Mathiesen EB, Waterloo K, Joakimsen O, Bakke SJ, Jacobsen EA and Bønaa KH (2004) Reduced neuropsychological test performance in asymptomatic carotid stenosis: The Tromsø Study. Neurology 62:695–701. doi: 10.1212/01.wnl.0000113759.80877.1f 15007116

[55] Georgiadis D, Sievert M, Cencetti S, Uhlmann F, Krivokuca M, Zierz S, et al. (2000) Cerebrovascular reactivity is impaired in patients with cardiac failure. Eur Heart J 21:407–13. doi: 10.1053/euhj.1999.1742 10666355

[56] Gruhn N, Larsen FS, Boesgaard S, Knudsen GM, Mortensen SA, Thomsen G et al. (2001) Cerebral blood flow in patients with chronic heart failure before and after heart transplantation. Stroke 32:2530–3. doi: 10.1161/hs1101.098360 11692012

[57] Nifosì F, Toffanin T, Follador H, Zonta F, Padovan G, Pigato G, et al. (2010) Reduced right posterior hippocampal volume in women with recurrent familial pure depressive disorder. Psychiatry Res 184:23–8. doi: 10.1016/j.pscychresns.2010.05.012 20817488

[58] Gottlieb SS, Khatta M, Friedmann E, Einbinder L, Katzen S, Baker B, et al. (2004) The influence of age, gender, and race on the prevalence of depression in heart failure patients. J Am Coll Cardiol 43:1542–9. doi: 10.1016/j.jacc.2003.10.064 15120809

[59] Gur RC, Ragland JD, Mozley LH, Mozley PD, Smith R, Alavi A, et al. (1997) Lateralized changes in regional cerebral blood flow during performance of verbal and facial recognition tasks: correlations with performance and "effort". Brain Cogn 33:388–414. doi: 10.1006/brcg.1997.0921 9126402

[60] Looi JC and Sachdev PS (2000) Vascular dementia as a frontal subcortical system dysfunction. Psychol Med 30:997–1003. doi: 10.1017/s003329179900269x 12027059

[61] Horstmann A, Frisch S, Jentzsch RT, Müller K, Villringer A and Schroeter ML (2010) Resuscitating the heart but losing the brain: brain atrophy in the aftermath of cardiac arrest. Neurology 74:306–12. doi: 10.1212/WNL.0b013e3181cbcd6f 20101036

[62] Woo MA, Macey PM, Fonarow GC, Hamilton MA and Harper RM (2003) Regional brain gray matter loss in heart failure. J Appl Physiol (1985) 95:677–84. doi: 10.1152/japplphysiol.00101.2003 12716866

[63] Kim MS, Lee GH, Kim YM, Lee BW, Nam HY, Sim UC, et al. (2017) Angiotensin II Causes Apoptosis of Adult Hippocampal Neural Stem Cells and Memory Impairment Through the Action on AMPK-PGC1α Signaling in Heart Failure. Stem Cells Transl Med 6:1491–1503. doi: 10.1002/sctm.16-0382 28244243 PMC5689768

[64] Niitsu T, Iyo M and Hashimoto K (2012) Sigma-1 receptor agonists as therapeutic drugs for cognitive impairment in neuropsychiatric diseases. Curr Pharm Des 18:875–83. doi: 10.2174/138161212799436476 22288409

[65] Inestrosa NC and Arenas E (2010) Emerging roles of Wnts in the adult nervous system. Nat Rev Neurosci 11:77–86. doi: 10.1038/nrn2755 20010950

[66] Ito K, Hirooka Y and Sunagawa K (2013) Brain Sigma-1 Receptor Stimulation Improves Mental Disorder and Cardiac Function in Mice With Myocardial Infarction. Journal of Cardiovascular Pharmacology 62:222–228. doi: 10.1097/FJC.0b013e3182970b15 23615161

