# Supplementary material for: Abnormalities of hippocampus and frontal lobes in heart failure patients and animal models with cognitive impairment or depression: A systematic review
Source: PLoS One. 2022 Dec 9;17(12):e0278398. doi: 10.1371/journal.pone.0278398 (PMC9733898; doi:10.1371/journal.pone.0278398)
Supplement: S1 File — (DOCX) [file pone.0278398.s002.docx]

**Searching strategy of English database** (PubMed, Web of science, Embase, Cochrane)

**#1**: (Heart failure OR Cardiac Failure OR Heart Decompensation OR Decompensation, Heart OR Heart Failure, Right-Sided OR Heart Failure, Right Sided OR Right-Sided Heart Failure OR Right Sided Heart Failure OR Myocardial Failure OR Congestive Heart Failure OR Heart Failure, Congestive OR Heart Failure, Left-Sided OR Heart Failure, Left Sided OR Left-Sided Heart Failure OR Left Sided Heart Failure)

**#2**: (Hippocampus OR Hippocampus Proper OR Hippocampus Propers OR Proper, Hippocampus OR Propers, Hippocampus OR Ammon Horn OR Horn, Ammon OR Ammon's Horn OR Ammons Horn OR Horn, Ammon's OR Cornu Ammonis OR Hippocampal Formation OR Formation, Hippocampal OR Formations, Hippocampal OR Hippocampal Formations OR Schaffer Collaterals OR Schaffer Collateral OR Collateral, Schaffer OR Collaterals, Schaffer OR Subiculum OR Subiculums)

**#3**: (Frontal Lobe OR Frontal Lobes OR Lobe, Frontal OR Lobus Frontalis OR Frontal Cortex OR Cortex, Frontal OR Frontal Cortices)

**#4**: (Cognition Disorders OR Disorder, Cognition OR Disorders, Cognition OR Overinclusion)

**#5**: (Depression OR Depressions OR Depressive Symptoms OR Depressive Symptom OR Symptom, Depressive OR Symptoms, Depressive OR Emotional Depression OR Depression, Emotional OR Depressions, Emotional OR Emotional Depressions OR Depressive Disorder OR Depressive Disorders OR Disorder, Depressive OR Disorders, Depressive OR Neurosis, Depressive OR Depressive Neuroses OR Depressive Neurosis OR Neuroses, Depressive OR Depression, Endogenous OR Depressions, Endogenous OR Endogenous Depression OR Endogenous Depressions OR Depressive Syndrome OR Depressive Syndromes OR Syndrome, Depressive OR Syndromes, Depressive OR Depression, Neurotic OR Depressions, Neurotic OR Neurotic Depression OR Neurotic Depressions OR Melancholia OR Melancholias OR Unipolar Depression OR Depression, Unipolar OR Depressions, Unipolar OR Unipolar Depressions)

**#6: ((#2) OR (#3))**

**#7: ((#4) OR (#5))**

**#8: ((#1) AND (#6)) AND (#7)**
